# Molecular probes for the identification of avian *Haemoproteus* and *Leucocytozoon* parasites in tissue sections by chromogenic *in situ* hybridization

**DOI:** 10.1186/s13071-019-3536-2

**Published:** 2019-06-03

**Authors:** Tanja Himmel, Josef Harl, Anna Kübber-Heiss, Cornelia Konicek, Nuhacet Fernández, Carles Juan-Sallés, Mikas Ilgūnas, Gediminas Valkiūnas, Herbert Weissenböck

**Affiliations:** 1 Institute of Pathology, Department of Pathobiology, University of Veterinary Medicine Vienna, Vienna, Austria; 2 Research Institute of Wildlife Ecology, Department of Integrative Biology and Evolution, University of Veterinary Medicine Vienna, Vienna, Austria; 3 Clinical Unit of Internal Medicine Small Animals, University of Veterinary Medicine Vienna, Vienna, Austria; 4 Loro Parque Fundación, Puerto de la Cruz, Spain; 5 Noah’s Path, Elche, Spain; 6 Nature Research Centre, Vilnius, Lithuania

**Keywords:** *In situ* hybridization, *Haemoproteus*, *Leucocytozoon*, Birds, Pathology, Exo-erythrocytic meronts, *18S* ribosomal RNA, Haemosporidians

## Abstract

**Background:**

Avian haemosporidian parasites can cause severe disease in their hosts due to excessive exo-erythrocytic merogony and anaemia caused by blood stages. Notably, the development of megalomeronts by species of *Haemoproteus* and *Leucocytozoon* has been associated with mortalities in birds. Diagnosis of lethal infections is currently accomplished by the detection of parasites’ tissue stages in histological sections combined with PCR and sequencing. However, sequences frequently are not reliably obtained and the generic discrimination of exo-erythrocytic tissue stages based on morphological characters is challenging. Therefore, the present study aimed at developing specific molecular probes for the identification of *Haemoproteus* spp. and *Leucocytozoon* spp. in histological sections using chromogenic *in situ* hybridization.

**Methods:**

Parasite subgenus-specific oligonucleotide probes were designed to target the *18S* ribosomal RNA of *Haemoproteus* species (subgenus *Parahaemoproteus*) and *Leucocytozoon* spp. (subgenus *Leucocytozoon*) and were *in situ* hybridized to sections from formalin-fixed, paraffin-embedded tissue samples determined positive for these parasites by PCR and histopathology. To confirm the presence of parasites at sites of probe hybridization, consecutive sections were stained with haematoxylin–eosin and examined.

**Results:**

*Parahaemoproteus*- and *Leucocytozoon*-specific probes labelled erythrocytic and exo-erythrocytic stages of *Haemoproteus* spp. and *Leucocytozoon* spp., respectively. Binding of probes to parasites was consistent with detection of the same exo-erythrocytic meronts in consecutive haematoxylin–eosin-stained sections. Cross-reactivity of the probes was ruled out by negative chromogenic *in situ* hybridization when applied to samples positive for a parasite of a genus different from the probes’ target.

**Conclusions:**

Chromogenic *in situ* hybridization using *18S* ribosomal RNA-specific oligonucleotide probes reliably identifies and discriminates *Haemoproteus* and *Leucocytozoon* parasites in tissue sections and enables unequivocal diagnosis of haemosporidioses.

**Electronic supplementary material:**

The online version of this article (10.1186/s13071-019-3536-2) contains supplementary material, which is available to authorized users.

## Background

Avian haemosporidian parasites (Haemosporida) are widespread almost all over the world and infect birds of diverse families and orders. The majority of described parasites belong to the genera *Plasmodium*, *Haemoproteus* and *Leucocytozoon*, comprising more than 200 morphologically distinct species [1]. The life-cycle of haemosporidians in the avian host includes development both in tissues (exo-erythrocytic merogony) and blood cells. Haemosporidians always undergo exo-erythrocytic merogony producing meronts in tissue cells before they infect blood cells and form gametocytes, the parasite stage infective for vectors [2].

The pathogenic impact of haemosporidians on the host can be variable at different developmental stages, and severity of infection in avian hosts depends on many factors [3]. Generally, haemosporidian infection is subclinical in birds; however, it is well established that parasites not only cause haematological disorders but can also elicit severe damage to organs due to marked merogony in tissues [2]. For example, *Plasmodium* infections can result in excessive multiplication of parasites in various organs including the brain, where meronts cause blockage of capillaries, ultimately leading to death of the host [4]. Such lethal infections were frequently reported in immunologically naive hosts, which are highly susceptible to infection, e.g. penguins [5–7]. However, there are also cases, albeit less numerous, which demonstrate mortalities linked to malaria in wild birds inhabiting regions with haemosporidian transmission [8–10]. Exo-erythrocytic merogony of *Haemoproteus* and *Leucocytozoon*, the most diverse avian haemosporidian genera besides *Plasmodium*, has been investigated only fragmentarily because of methodological difficulties to access these life-cycle stages, particularly in wild animals. Data on complete tissue merogony are available only for a few of these blood parasite species [2]. Experimental research might provide convincing data [1], but is challenging to design with *Haemoproteus* and *Leucocytozoon* parasites, mainly because of difficulties to obtain sporozoites, the only infective stage that can initiate exo-erythrocytic development of these haemosporidians in vertebrate hosts. Cultivation and experimental infection of vectors (species of Ceratopogonidae, Hippoboscidae and Simuliidae) are needed for such experiments. With rare exceptions, *Haemoproteus* and *Leucocytozoon* parasites were usually considered to be relatively benign in avian hosts, particularly in wildlife [11]. That hampered research in this field of parasitology and veterinary medicine for better understanding virulence of haemosporidian infections. Numerous veterinary observations and field case reports contradicted this opinion, but they did not receive due attention [12–16]. *Haemoproteus* and *Leucocytozoon* parasites have been identified to cause morbidity and mortality in several bird species. Severe haemosporidioses caused by these parasites have been repeatedly reported in both captive [12–15, 17–25] and wild birds [9, 10, 16, 18, 26] and were commonly associated with the development of megalomeronts in diverse organs of their hosts.

Unequivocal determination of parasitic tissue stages in haemosporidian infections is difficult. Diagnosis of haemosporidian infections in lethal cases is primarily achieved by necropsy followed by histopathological examination and PCR-based testing. Parasites can be detected in routinely stained histological sections; however, microscopic examination is time-consuming and parasites can be overlooked or confounded with nuclear fragments in necrotic tissue or cell debris. Although morphological features can indicate their generic identity, the correct assignment of parasites is often challenging, especially in cases of aberrant infections due to unusual morphology and location in atypical hosts [2]. There are several reports presenting severe cases of haemosporidioses, in which conclusive determination of the parasites based on tissue stages was not achieved, particularly in cases of *Haemoproteus* and *Leucocytozoon* infections [10, 26–28]. In many cases, identification of involved parasites is accomplished by histological analysis in combination with PCR and sequencing. However, even sensitive PCR does not always reliably detect parasites, especially in co-infections, which are common in birds [29] hampering correct attribution of detected genotypes to morphological stages observed in histology. This is a particularly sensitive issue in distinguishing growing (young) exo-erythrocytic meronts of *Plasmodium* and *Haemoproteus* parasites, which usually are morphologically identical at this stage of development [1].

The aim of the present study was to develop molecular probes for the specific detection of *Haemoproteus* (subgenus *Parahaemoproteus*) and *Leucocytozoon* (subgenus *Leucocytozoon*) parasites in formalin-fixed, paraffin wax-embedded (FFPE) tissue samples by using chromogenic *in situ* hybridization (CISH). This method has already been established for the detection of *Plasmodium* species by using probes which target the *18S* ribosomal RNA (rRNA) of parasites [7]. Based on these convincing results, molecular probes targeting the *18S* rRNA of *Haemoproteus* species and *Leucocytozoon* spp. were designed.

## Methods

### Probe design

Oligonucleotide probes were designed to target the *18S* rRNA of parasites from the avian haemosporidian genera *Haemoproteus* (subgenus *Parahaemoproteus*) and *Leucocytoz*oon (subgenus *Leucocytozoon*). Haemosporidian *18S* ribosomal DNA (rDNA) sequences were isolated from genomes published in NCBI GenBank [30] (accession numbers X13706, AY625607, PRJEB9073, PRJEB9074) and aligned with sequences obtained from an analysis of nuclear *18S* rDNA of a selection of *Haemoproteus* spp., *Leucocytozoon* spp. and *Plasmodium* spp. (JH, unpublished data) using MAFFT v. 7 [31]. Alignments were checked in Bioedit [32] and regions of sequence homology suitable for parasite subgenus-specific probes, i.e. conserved within one target group but with differences (mismatches) to other groups, were determined. Probe design was conceived to address species of the subgenera *Parahaemoproteus* (*Haemoproteus*) and *Leucocytozoon* (*Leucocytozoon*), as they comprise the majority of avian *Haemoproteus* and *Leucocytozoon* species.

For the detection of *Haemoproteus* (*Parahaemoproteus*) species, the probe P-Haemo18S_1 (5′-CTA ACC GTA GTT ATA GTC GCC ATC TC-3′) was designed. This probe is 100% complementary to the *18S* rRNA of *Haemoproteus minutus* (TURDUS2), *H. syrnii* (CULKIB01), *H. lanii* (RBS06), *H. balmorali* (ROBIN01), *H. tartakovskyi* (SISKIN1), and the *Haemoproteus* sp. lineages STAL2, LK03 and EMCIR01. Because of substantial *18S* rDNA sequence differences between *Leucocytozoon* species belonging to the *Leucocytozoon toddi*-species complex and other *Leucocytozoon* spp. (JH, unpublished data), two distinct probes were designed to target parasites of the subgenus *Leucocytozoon*: the probe Leuco18S_1 (5′-TAG GAC TCC CCA CTT GTC TTT TTC TTG A-3′), which is 100% complementary to the *18S* rRNA of *Leucocytozoon* sp. lineages CIAE02, SYCON05, TUPHI06, PARUS20, COCOR18, STAL05, BUBO01, ASOT06, MILVUS02, COCOR9, BT1, COCOR13 and STAL3, and the probe Ltod18S_1 (5′-GCT AAC CGT AGT TAT AGT CGC CAT CTC-3′), which is 100% complementary to *Leucocytozoon* sp. lineages ACNI03, CIAE03 and BUTBUT03, and targets members of the *L. toddi*-species complex. To ensure specificity of probes, they were designed to include at least six nucleotide mismatches to *18S* rRNA sequences of other target groups. Quality, i.e. GC-content, formation of self-dimers, and hybridization temperature of probes was checked *in silico* using AmplifX v. 1.7 [33], and a BLAST search against bird genomes was performed in NCBI GenBank to exclude unintentional hybridization to bird DNA. Probes were commercially synthesized by Microsynth Austria (Vienna, Austria) including 3′ ends labelled with digoxigenin.

### Tissue samples

Formalin-fixed, paraffin wax-embedded (FFPE) tissue samples including various organs from infected wild birds were obtained from the archive of the Institute of Pathology and the Research Institute of Wildlife Ecology at the University of Veterinary Medicine Vienna (Austria). For testing the new probes, only samples determined as monoinfections by PCR were used. Tissue samples were sectioned at 1 µm, mounted on SuperFrost Plus Slides (Thermo Fisher Scientific, Waltham, Massachusetts, USA) and air-dried before being subjected to haematoxylin–eosin (HE) staining or CISH. To be able to precisely correlate histological findings with CISH results, serial sections were prepared, one of which was stained with HE and the others used for CISH with each single probe. Probes were tested on a selection of samples positive for different lineages in order to evaluate applicability of probes on a subgenus level (Table 1).

**Table 1:** Host species and identified lineages from samples used for testing subgenus-specific probes

Host	Parasite	MalAvi lineage	GenBank ID
*Asio otus*	*Haemoproteus* sp.	CIRCUM06	MK330165
*Strix uralensis*	*Haemoproteus* sp.	STRURA03	MK330140
*Strix uralensis*	*H. syrnii*	CULKIB01	MK330143, MK330144
*Tetrao urogallus*	*Haemoproteus* sp.	TETURO01	MK330141
*Tetrao urogallus*	*Haemoproteus* sp.	TETURO02	MK330139
*Cyanoramphus novaezelandiae*	*H. minutus*	TUPHI01	MK330147
*Cyanoramphus novaezelandiae*	*H. minutus*	TURDUS2	MK330158
*Turdus merula*	*H. minutus*	TURDUS2	MK330151
*Passer domesticus*	*H. lanii*	RBS06	MK330149
*Lanius collurio*	*H. lanii*	RBS2	MK330164
*Emberiza citrinella*	*Haemoproteus* sp.	EMCIR01	MK330150, MK330152
*Falco tinnunculus*	*Haemoproteus* sp.	LK03	MK330145, MK330146
*Sylvia atricapilla*	*H. parabelopolskyi*	SYAT41	MK330159
*Passer domesticus*	*Haemoproteus* sp.	PADOM03	MK330161, MK330162, MK330163
*Bubo bubo*	*Leucocytozoon danilewskyi*	BUBO01	MK330142
*Corvus corone cornix*	*Leucocytozoon* sp.	COCOR13	MK330138
*Turdus merula*	*Leucocytozoon* sp.	ASOT06	MK330148
*Garrulus glandarius*	*Leucocytozoon* sp.	GAGLA06	MK330153
*Turdus merula*	*Leucocytozoon* sp.	TUMER10	MK330154
*Brotogeris cyanoptera*	*Leucocytozoon* sp.	CIAE02	MK330160
*Buteo buteo*	*L. toddi*	BUTBUT03	MK330155
*Turdus merula*	*Plasmodium matutinum*	LINN1	MK330156, MK330157

### Molecular analysis

Molecular analysis was performed for all samples that were used for probe design and CISH. Total DNA was extracted from frozen and FFPE tissue samples and, if available, also from dried blood spots. For frozen samples and dried blood spots the DNeasy Blood & Tissue Kit (Qiagen, Venlo, Netherlands), and for FFPE tissue samples the FFPE Kit (Qiagen) were used according to the manufacturer’s recommendations with the exception that two elutions with each 100 µl AE buffer were performed, the second of which was used as template for the PCR. Samples were screened for haemosporidians using a nested PCR protocol [34] targeting a section of the mitochondrial cytochrome *b* gene (*cytb*) of parasites. In the first PCR, the primers HaemNFI and HaemNR3 were used to amplify parasite mitochondrial DNA of *Plasmodium* spp., *Haemoproteus* spp. and *Leucocytozoon* spp. In the second PCR, the primer pairs HaemF/HaemR2 and HaemFL/HaemR2L were used to amplify mitochondrial DNA of *Plasmodium* spp./*Haemoproteus* spp. and *Leucocytozoon* spp., respectively. Each DNA extract was amplified once. The primers of Hellgren et al. [34] do not allow for amplification of *cytb* sequences in members of the *L. toddi-*species complex, which are primarily found in raptors belonging to the order Accipitriformes. Based on the alignment of *cytb* sequences from species of the *L. toddi-*species complex published on NCBI GenBank under the accession numbers DQ177235–DQ177273 [35] and HM142915–HM142923 [36], a nested PCR protocol was designed to specifically target this group: in the first PCR, the primers CytB_L2_F (5′-GAG AGT TAT GGG CTG GAT GGT-3′) and CytB_L2_R (5′-TAG AAA GCC AAG AAA TAC CAT TCT G-3′), and in the second PCR, the primers CytB_L2_nF (5′-GCT GGA TGG TGT TTT AGA TAY ATG C-3′) and CytB_L2_nR (5′-CCA TTC TGG AAC AAT ATG TAA AGG TG-3′) were used to amplify a 528 bp section of the *cytb* from respective parasites. The selected section covers the entire *cytb* barcoding region, which is amplified using the protocol of Hellgren et al. [34].

PCR was performed using the GoTaq G2 Flexi DNA Polymerase (Promega, Madison, Wisconsin, USA). The volume for each reaction mixture was 25 µl and contained 14.375 µl nuclease-free water, 5 µl 5× Green GoTaq Flexi Buffer, 2 µl MgCl_2_ solution (25 mM), 0.5 µl PCR nucleotide mix (10 mM, Promega), 0.125 µl GoTaq G2 Flexi DNA Polymerase (5 u/µl), each 1 µl forward and reverse primer (10 pmol/µl) and 1 µl of DNA template. For the second PCR, instead of a DNA template, 1 µl of the PCR product from the first PCR was used. Thermocycler conditions were the following: initial denaturation at 94 °C for 2 min, 35 cycles of denaturation at 94 °C for 30 s, primer annealing at 50 °C (Hellgren primers)/55 °C (*L. toddi* primers) for 30 s, and extension at 72 °C for 1 min, followed by a final extension at 72 °C for 10 min. PCR products were run on 1% agarose gels stained with Midori Green Advance (Nippon Genetics Europe, Dueren, Germany) and visualized with a BioSens SC-Series 710 gel documentation system (GenXpress, Wiener Neudorf, Austria). All DNA extracts were amplified once. Tissue samples confirmed positive in earlier screenings were used as positive controls. Negative controls contained nuclease free water instead of DNA templates. PCR amplicons from the second PCRs were sent to Microsynth Austria for bi-directional sequencing with forward and reverse primers. Obtained sequences were edited and aligned in Bioedit [32] and electropherograms were carefully checked for double base-calling to rule out mixed infections. Sequences were submitted to BLAST search in the avian malaria database MalAvi [37] and NCBI GenBank.

### Chromogenic *in situ* hybridization

CISH was applied to tissue sections following a previously established protocol [7] with some modifications. Tissue sections were dewaxed 2 × 5 min in Neo-Clear solution (Merck Millipore, Burlington, Massachusetts, USA), rehydrated in a series of 100%, 90% and 70% ethanol and distilled water 5 min each and subjected to proteolytic treatment with proteinase K (Roche, Basel, Switzerland) 3 µg/ml in 0.5 M Tris-HCl buffered saline at 37 °C for 40 min. After proteolysis, sections were rinsed in distilled water, dehydrated in 90% and 100% ethanol for 5 min and air-dried. Tissue sections were covered with a hybridization mixture containing 11 µl distilled water, 20 µl 20× saline-sodium citrate (SSC) buffer, 50 µl formamide, 5 µl herring sperm, 2 µl 50× Denhardt’s solution, 10 µl dextran sulphate (50%, w/v) and 1 ng probe per 100 µl. Slides were incubated at 95 °C for 6 min and shock-frozen on crushed ice, followed by incubation overnight in a humid chamber at 40 °C. All following steps were performed in a humid chamber at room temperature. First, sections were washed 3 × 10 min in 2×, 1× and 0.1× SSC buffer and pre-incubated with Tris-HCL buffered saline, normal goat serum and 10% Triton X-100 for 30 min before incubation with anti-digoxigenin-AP Fab-fragments (Roche) at a concentration of 1:200 for 1 h. After washing 2 × 15 min in distilled water, the chromogenic substrates NBT (nitro-blue tetrazolium chloride, Roche) and BCIP (5-bromo-4-chloro-3′-indolyphosphate p-toluidine salt, Roche) were mixed with levamisole in 0.1 M Tris-buffered saline (pH 9.5) and with applied to sections for a minimum of 40 min. Development of signal was examined by visual control and colour reaction was terminated by placing the sections in Tris-EDTA buffer (pH 8.0) for 10 min. Finally, sections were counterstained with haematoxylin and mounted using Aquatex (Merck Millipore) and coverslips.

In a first test, different concentrations of probes (0.25–1 ng/100 µl hybridization mixture) and colour reaction times (40–120 min) were applied to determine optimal working concentrations for each probe. After testing the two probes for *Leucocytozoon* species individually, they were combined in a hybridization-cocktail at optimal working concentration for each probe.

Subgenus-specificity of probes was tested by applying them on samples positive for parasites belonging to subgenera different from the probes’ targets, including a *Plasmodium* sp. positive sample. Conversely, a *Plasmodium*-specific probe [7] was applied to rule out co-infections which might have been missed by PCR screening. For negative controls, probes were omitted in the hybridization mixture. Furthermore, probes were tested for cross-hybridization with a range of other organisms including viral (West Nile virus, Usutu virus), fungal (*Aspergillus* sp., *Encephalitozoon cuniculi*) and protozoal (*Eimeria* sp., *Cryptosporidium* sp., *C*. *tortoise*, *Toxoplasma* sp., *Babesia* sp., *Tetratrichomonas gallinae*, *Histomonas meleagridis*, *Entamoeba* sp., *Blastocystis* sp.) pathogens.

### Microscopy

Haematoxylin–eosin stained sections and CISH results were evaluated by bright field microscopy using 200×, 400× and 1000× magnifications. Images were acquired using an Olympus BX51 microscope (Olympus Europa, Hamburg, Germany) equipped with an Olympus DP71 camera. Images were adjusted for brightness and contrast and assembled in Adobe Photoshop CC 2015 (Adobe, San José, California, USA).

## Results

### Molecular analysis

Samples used in this study were determined as single infections by nested PCR. Sequence electropherograms did not show any double peaks indicating absence of co-infections in the samples. Detected *cytb* sequences were submitted to BLAST search in NCBI GenBank and MalAvi [37]. Most sequences were 100% identical with already published lineages (Table 1). Five new haplotypes were identified in samples that were used for testing the probes (STRURA03, TETURO01, TETURO02, TUMER10, BUTBUT03), and were consequently submitted to MalAvi [37]. Nucleotide sequences were deposited in NCBI GenBank under the accession numbers MK330138–MK330165 (Table 1).

### Detection of erythrocytic and exo-erythrocytic parasite stages by CISH

**Fig. 1:**
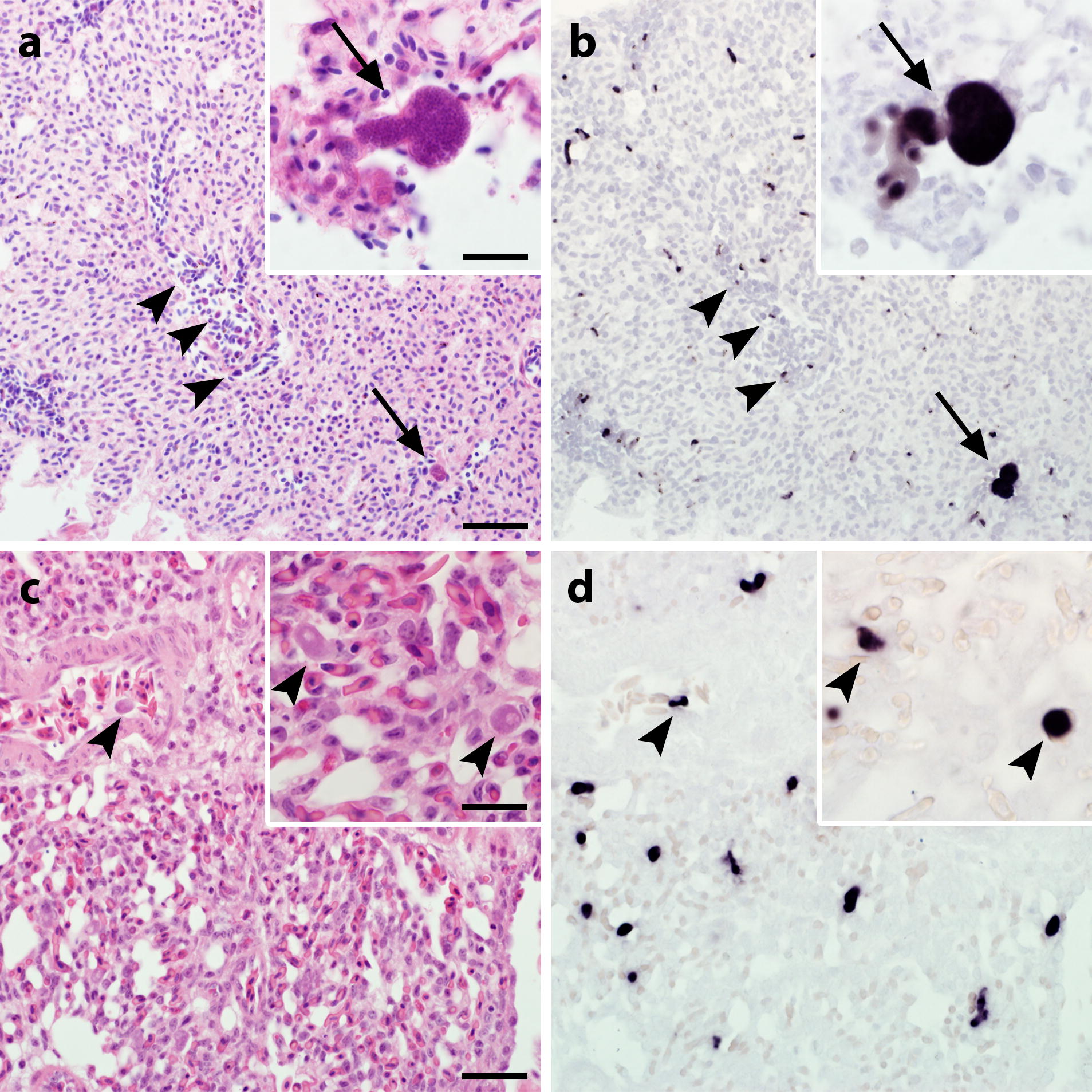
Erythrocytic and exo-erythrocytic parasite stages in lung sections from infected birds visualized by chromogenic *in situ* hybridization (CISH). Serial lung sections from a yellowhammer (*Emberiza citrinella*) infected with *Haemoproteus* sp. lineage EMCIR01 (**a**, **b**) and a crow (*Corvus cornix*) infected with *Leucocytozoon* sp., lineage COCOR13 (**c**, **d**) were stained with haematoxylin–eosin (HE; **a**, **c**) and subjected to CISH (**b**, **d**). Gametocytes (arrowheads) and exo-erythrocytic meronts (arrows) of parasites were observed in HE-stained sections (**a**, **c**) and labelled by subgenus-specific probes for *Haemoproteus* (*Parahaemoproteus*) spp. (**b**) and *Leucocytozoon* (*Leucocytozoon*) spp. (**d**) showing distinct purple signals in corresponding *in situ*-hybridized sections. Note that small parasites are difficult to see in HE-stained sections, but are readily visible in sections subjected to CISH. *Scale-bars*: 20 µm (inserts, 10 µm)

*Haemoproteus* and *Leucocytozoon* parasites were successfully visualized by CISH using parasite subgenus-specific probes in positive tissue samples. Parasite stages were labelled by distinct dark purple signals of the chromogenic reaction products when subgenus-specific probes matching the parasites’ rRNA in the sample were applied (Fig. 1b, d). Signals were observed in different organs, including lungs, liver, spleen, brain, intestine, heart and skeletal muscle. The abundance and intensity of signals varied between samples and organs depending on the distribution of parasite stages. Purple signals were discernible in blood cells of capillaries and larger vessels indicating labelling of erythrocytic and leucocytic parasite stages of *Haemoproteus* spp. and *Leucocytozoon* spp. Signals attributed to exo-erythrocytic meronts perfectly matched the parasites’ shapes and locations as observed in corresponding HE-stained sections. Application of probes not matching the parasites’ subgenera remained negative demonstrating high subgenus-specificity of probes (Additional file 1: Figure S1c, d, f, h, j, k).

### Labelling of megalomeronts of *Haemoproteus* and *Leucocytozoon* parasites in aberrant hosts

**Fig. 2:**
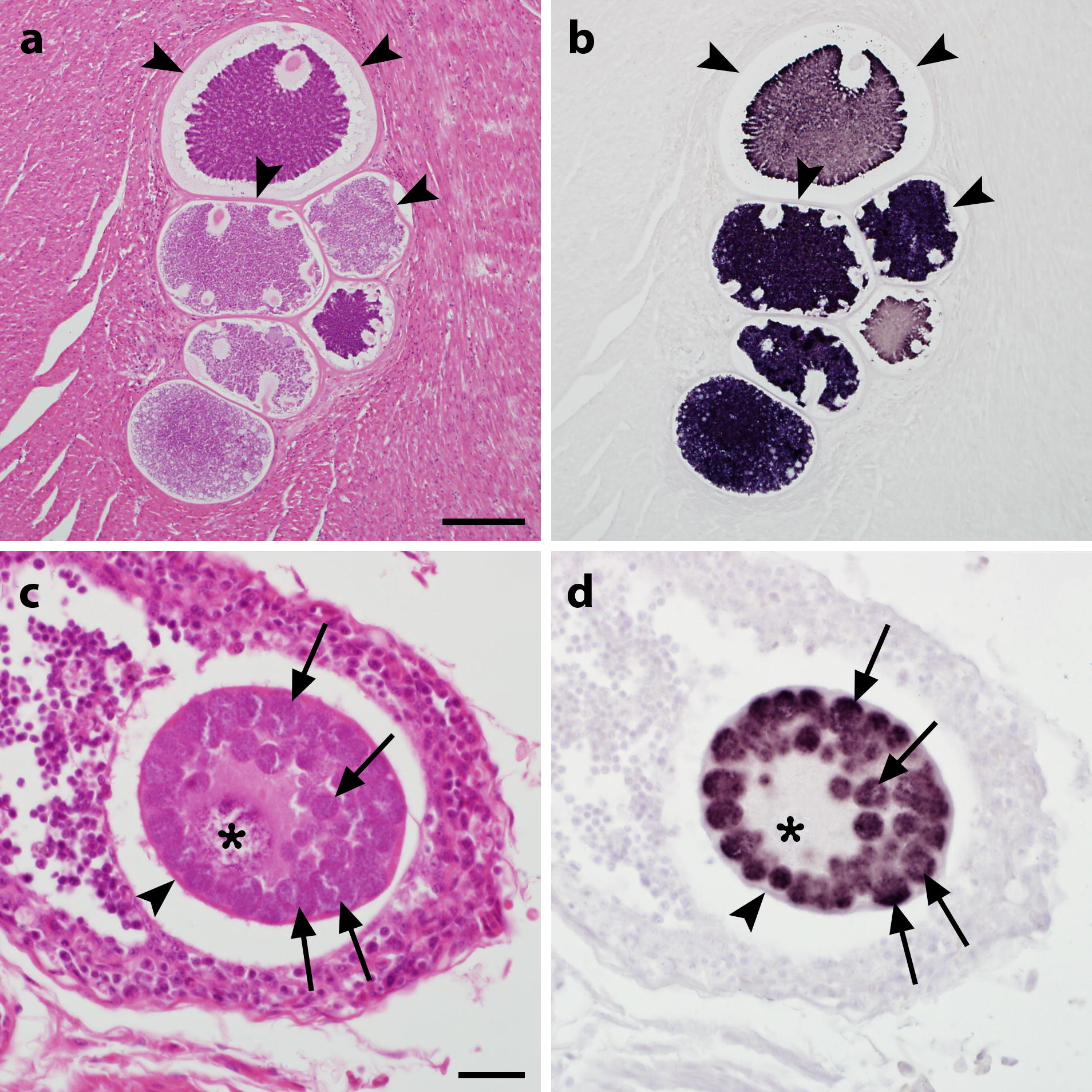
Megalomeronts of *Haemoproteus minutus* (**a**, **b**) and *Leucocytocoon* sp. (**c**, **d**) in histological sections from infected parakeets stained by haematoxylin and eosin (HE; **a**, **c**) and chromogenic *in situ* hybridization (CISH; **b**, **d**). Multiple megalomeronts of *H. minutus*, lineage TUPHI01, and *Leucocytozoon* cf. *californicus*, lineage CIAE02, were observed in HE-stained sections of the cardiac muscle in a red-crowned parakeet (*Cyanoramphus novaezelandiae*; **a**) and in the bursa of Fabricius in a blue-winged parakeet (*Brotogeris cyanoptera*; **c**), respectively. Application of subgenus-specific probes for *Haemoproteus* (*Parahaemoproteus*) spp. (**b**) and *Leucocytozoon* (*Leucocytozoon*) spp. (**d**) with CISH unequivocally indicated generic identity of parasites. Signals were confined to cytomeres (arrows) and merozoites of the parasites whereas structures not attributed to the parasite such as host cell nucleus (asterisk) and prominent capsule-like walls (arrowheads) around megalomeronts remained negative. Note numerous roundish cytomeres (arrows) and a readily visible host cell nucleus (or ‘central body’) present in the megalomeront of *Leucocytozoon* sp., but absent in megalomeronts of *Haemoproteus* parasites. *Scale-bars*: **a**, 100 µm; **c**, 20 µm

To investigate whether the probes also reliably bind to megalomeronts of *Haemoproteus* spp. and *Leucocytozoon* spp. in non-adapted hosts, three cases of aberrant infections were examined. Two of them were red-crowned parakeets (*Cyanoramphus novaezelandiae*) infected with *H. minutus*, lineages TUPHI01 and TURDUS2, and one was a cobalt-winged parakeet (*Brotogeris cyanoptera*) infected with *Leucocytozoon* cf. *californicus*, lineage CIAE02. *In situ* hybridization with subgenus-specific probes revealed robust labelling of megalomeronts in different tissues (Fig. 2b, d). Staining intensity varied between megalomeronts from different organs of the same sample, but also across megalomeronts found within the same organ (Fig. 2b). Similarly, colour variations in regard to the basophilic and eosinophilic nature of respective structures were observed in corresponding HE-stained sections (Fig. 2a). Hybridization of probes not complementary to the parasites’ genera remained negative (Additional file 2: Figure S2c, d, f, h).

### Cross-reactivity testing

*In situ* hybridization failed to produce signals in tissue samples positive for different viral, fungal and protozoal organisms ruling out undesirable binding of probes with pathogens which potentially might co-occur in infected birds.

## Discussion

In the present study, oligonucleotide probes were developed for the identification of *Haemoproteus* (*Parahaemoproteus*) species and *Leucocytozoon* (*Leucocytozoon*) spp. in FFPE tissues samples from infected birds. The results demonstrate that the new probes reliably detect erythrocytic and exo-erythrocytic parasite stages and distinguish between parasites belonging to different genera using CISH, providing opportunities to gain new knowledge about exo-erythrocytic development of haemosporidians using samples collected in the wild.

A similar methodological approach has already been successfully applied for detection of *Plasmodium* parasites in histological sections of infected birds [4, 7, 8]. A different CISH protocol using a *cytb* gene probe was used for the detection of *Haemoproteus* parasites [23]. However, in the latter study, the probe was designed to target the so-called barcode region of the mitochondrial *cytb* of *Haemoproteus* species and *Plasmodium* spp. and does not allow for discrimination between parasites of these genera. In the present study, probes for *Haemoproteus* species and *Leucocytozoon* spp. were designed to target the *18S* rRNA of parasites due to two major reasons. First, rRNA is highly abundant in the cell cytoplasm, improving detectability of parasites as compared to other target molecules. Secondly, the patchy conservation of the *18S* rRNA gene allowed for the design of subgenus-specific probes, which are necessary for the discrimination of parasites belonging to the most important avian haemosporidian genera.

Because *18S* rDNA sequences of *Haemoproteus* and *Leucocytozoon* parasites were not available in NCBI GenBank except for *H. tartakovskyi* [38], probe design was based on newly generated sequence data of *Haemoproteus* (*Parahaemoproteus*) spp. and *Leucocytozoon* (*Leucocytozoon*) spp. (JH, unpublished data). Acquired *18S* rDNA sequences and hence probe design included a relatively small selection of *Haemoproteus* and *Leucocytozoon* lineages (8 and 16, respectively), taking into account the huge number of reported parasite lineages from these subgenera. However, selected lineages represented diverse clades within both subgenera and the results demonstrated successful hybridization of the probes to lineages not included for probe design. Therefore, it can be presumed that the specified probes also detect other *Haemoproteus* and *Leucocytozoon* lineages, as selected target regions of the *18S* rRNA gene seem to be homologous across the majority of clades within the subgenus *Parahaemoproteus* and the two clades of the subgenus *Leucocytozoon*.

Despite evident advantages of targeting rRNA, some limitations may arise with respect to parasite detection by means of CISH when working with tissue samples that are not adequately fixed before being subjected to molecular analysis. Weak or false negative results may be obtained if tissues are poorly fixed, as this will result in RNA degradation. Conversely, extensive fixation over longer time periods (a month to years) leads to fragmentation or masking of nucleic acids and prevents hybridization of probes [39]. Ideally, tissue samples should be fresh and fixed immediately after necropsy to minimize autolytic degradation. Conventional fixation in 10% neutral buffered formalin for 12–72 hours should provide good morphology and detectability of rRNA by CISH.

Failure to detect parasites may also be the result of low cellular ribosome content, which could be the case in stages with reduced cellular activity, e.g. potential quiescent or dormant stages, similar to human malaria parasites [40]. Therefore, CISH results should always be carefully evaluated and compared to HE-stained sections to confirm absence of parasites.

The present study showed varying abundance and intensity of CISH signals between samples and different organs of individual samples, depending on parasite load and stage of infection. While some cases exhibited signals exclusively in blood cells, others presented both labelled erythrocytic and exo-erythrocytic stages. It might be the case that sparsely distributed meronts in the test samples were missed by investigation of only few sections during probe testing. The scope of the present study was to establish molecular probes rather than detailed investigation of the test samples; however, it should be stressed, that multiple sections must be examined before drawing conclusions on parasite load and presence of tissue meronts in general.

Megalomeronts detected in *Haemoproteus-* and *Leucocytozoon*-infected tissue samples showed different staining intensities in both *in situ*-hybridized and corresponding HE-stained sections. These variations possibly reflect different developmental stages of megalomeronts exhibiting variable levels of rRNA content; however, this assumption remains speculative.

This study proves that the clusters of megalomeronts (or so called multilocular megalomeronts) covered by thick walls (Fig. 2a, b) belong to *Haemoproteus* parasites, as suggested by Earlé et al. [14]. These parasites were formerly believed to be stages of development of *Leucocytozoon* species or even *Besnoitia* parasites [11]. However, it remains unclear whether multilocular megalomeronts develop only in non-adapted (aberrant) avian hosts or they are an essential stage of development of *Haemoproteus* species in natural hosts as well. During this study, only small meronts were reported in naturally infected yellowhammers, a natural host of *Haemoproteus* sp. lineage EMCIR01 (Fig. 1a, b). Application of CISH provides an opportunity to answer this question, which is important for better understanding mechanisms of virulence during haemoproteosis.

In addition to traditional histological methods for the diagnosis of haemosporidian infections, CISH can be used to localize and identify *Haemoproteus* spp. and *Leucocytozoon* spp. in tissue sections. A major benefit of CISH is that it enables specific parasite detection in morphologically preserved tissues and simultaneous evaluation of potentially associated lesions. This is particularly important considering numerous reports on the pathogenic effects that exo-erythrocytic parasite stages can have on their hosts [4, 7, 9, 10, 18, 22–26]. The correct determination of parasites associated with severe microscopic lesions is crucial for gaining a better understanding on the pathogenicity of diverse avian haemosporidian parasites. The methodology and probes presented here provide a suitable approach for the unequivocal generic assignment of *Haemoproteus* spp. and *Leucocytozoon* spp., facilitating the diagnosis of avian haemosporidioses in histological samples. Direction of future research will be the design of probes allowing identification of parasites at species level, an application which will enable new insights into tissue merogony of haemosporidians in mixed infections.

## Conclusions

This study demonstrates that *Haemoproteus* and *Leucocytozoon* parasite tissue stages can be readily detected and discriminated by CISH using *18S* rRNA-specific probes. In addition to the formerly established *Plasmodium*-specific probe, the new probes for *Haemoproteus* species and *Leucocytozoon* spp. represent powerful tools not only for diagnostic purposes but also for the study of exo-erythrocytic merogony of haemosporidian parasites in general. Further development of the *in situ* hybridization protocol towards the combined application of the probes will be useful to investigate co-infections, which predominate in birds, but have not been addressed from the histological point of view as far.

## Additional files

**Additional file 1: Figure S1.** Erythrocytic and exo-erythrocytic parasite stages in lung sections from infected birds visualized by chromogenic *in situ* hybridization (CISH). Serial lung sections from *Emberiza citrinella* infected with *Haemoproteus* sp. (EMCIR01) (**a**–**d**), *Corvus cornix* infected with *Leucocytozoon* sp. (COCOR13) (**e**–**h**) and *Turdus merula* infected with *Plasmodium matutinum* (LINN1) (**i**–**l**) were stained with haematoxylin–eosin (HE; **a**, **e**, **i**) and subjected to CISH with probes for *Haemoproteus* (*Parahaemoproteus*) spp. (**b**, **f**, **j**), *Leucocytozoon* (*Leucocytozoon*) spp. (**c**, **g**, **k**) and *Plasmodium* spp. (**d**, **h**, **l**). Blood stages (arrowheads) and exo-erythrocytic meronts (arrows) of parasites were observed in HE-stained sections (**a**, **e**, **i**) and labelled in corresponding *in situ*-hybridized sections (**b**, **g**, **l**). Sections treated with probes not matching the parasites’ genera, remained negative (**c**, **d**, **f**, **h**, **j**, **k**). *Scale-bars*: 20 µm (inserts: 10 µm).

**Additional file 2: Figure S2.** Megalomeronts of *Haemoproteus minutus* (**a**–**d**) and *Leucocytocoon* sp. (**e**–**h**) in histological sections from infected parakeets stained by haematoxylin-eosin (HE; **a**, **e**) and chromogenic *in situ* hybridization (CISH; **b**–**d**, **f**–**h**). Multiple megalomeronts of *Haemoproteus minutus* (TUPHI01), and *Leucocytozoon* cf. *californicus* (CIAE02), were observed in HE-stained sections of the cardiac muscle of *Cyanoramphus novaezelandiae* (**a**) and in the bursa of Fabricius of *Brotogeris cyanoptera* (**c**). CISH with probes for *Haemoproteus* (*Parahaemoproteus*) spp. (**b**, **f**), *Leucocytozoon* (*Leucocytozoon*) spp. (**c**, **g**) and *Plasmodium* spp. (**d**, **h**) indicated generic identity of parasites. Signals were confined to cytomeres (arrows) and merozoites of the parasites whereas host tissue structures like nucleus (asterisk) and capsule-like walls (arrowheads) around megalomeronts remained negative. Note numerous cytomeres (arrows) and a host cell nucleus present in the megalomeront of *Leucocytozoon* sp., but absent in megalomeronts of *Haemoproteus* parasites. Sections treated with probes not matching the parasites’ genera were negative (**c**, **d**, **f**, **h**). *Scale-bars*: **a**, 100 µm; **e**, 20 µm.

## Data Availability

The dataset supporting the conclusions of this article is included within the article and its additional files.
