# Supplementary figures and images for: Molecular probes for the identification of avian Haemoproteus and Leucocytozoon parasites in tissue sections by chromogenic in situ hybridization
Source: Parasit Vectors. 2019 Jun 3;12:282. doi: 10.1186/s13071-019-3536-2 (PMC6547609; doi:10.1186/s13071-019-3536-2)

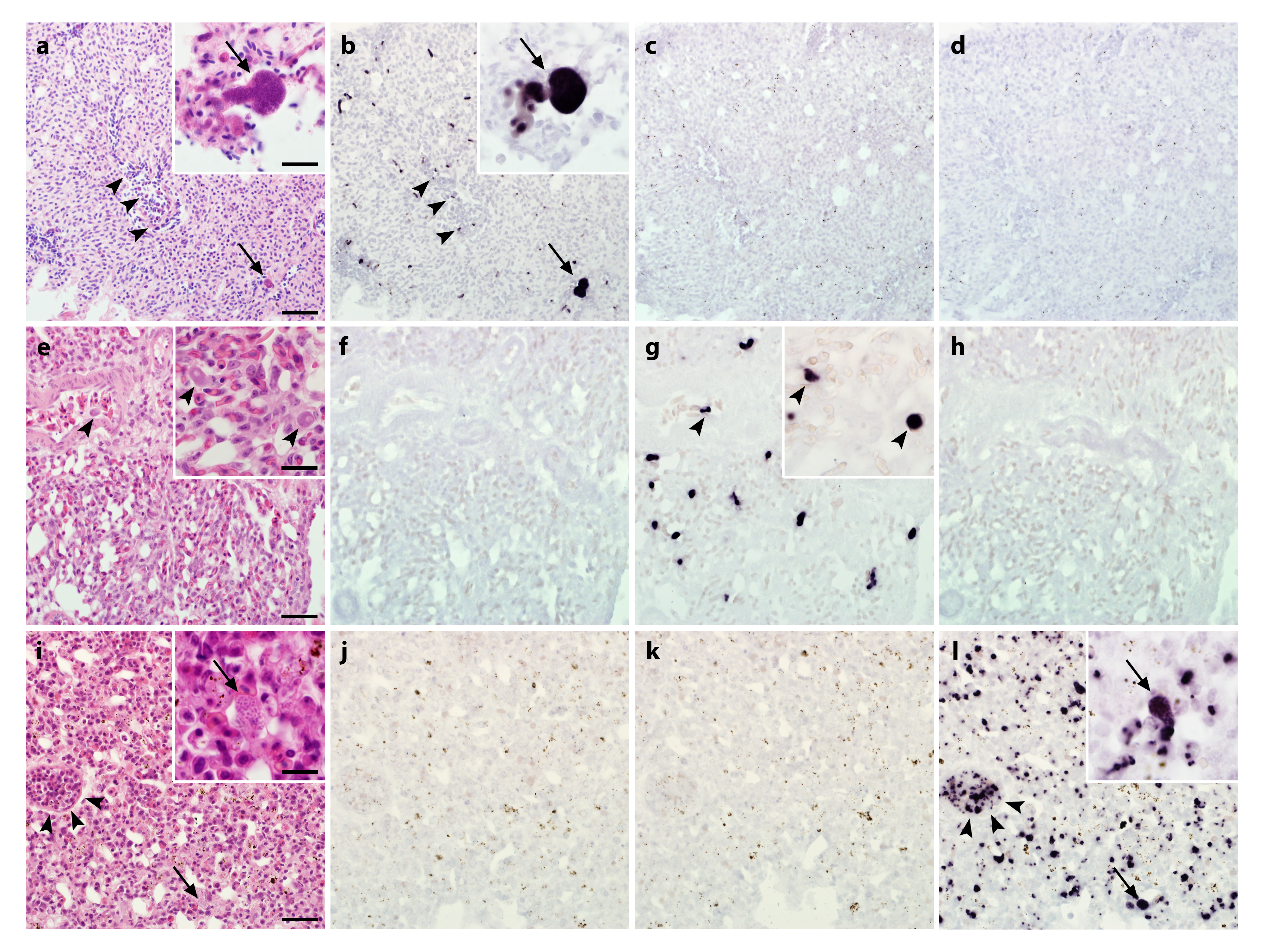

Supplement: Supplementary file 1 — Additional file 1: Figure S1. Erythrocytic and exo-erythrocytic parasite stages in lung sections from infected birds visualized by chromogenic in situ hybridization (CISH). Serial lung sections from Emberiza citrinella infected with Haemoproteus sp. (EMCIR01) (a–d), Corvus cornix infected with Leucocytozoon sp. (COCOR13) (e–h) and Turdus merula infected with Plasmodium matutinum (LINN1) (i–l) were stained with haematoxylin–eosin (HE; a, e, i) and subjected to CISH with probes for Haemoproteus (Parahaemoproteus) spp. (b, f, j), Leucocytozoon (Leucocytozoon) spp. (c, g, k) and Plasmodium spp. (d, h, l). Blood stages (arrowheads) and exo-erythrocytic meronts (arrows) of parasites were observed in HE-stained sections (a, e, i) and labelled in corresponding in situ-hybridized sections (b, g, l). Sections treated with probes not matching the parasites’ genera, remained negative (c, d, f, h, j, k). Scale-bars: 20 µm (inserts: 10 µm). [file 13071_2019_3536_MOESM1_ESM.tif]

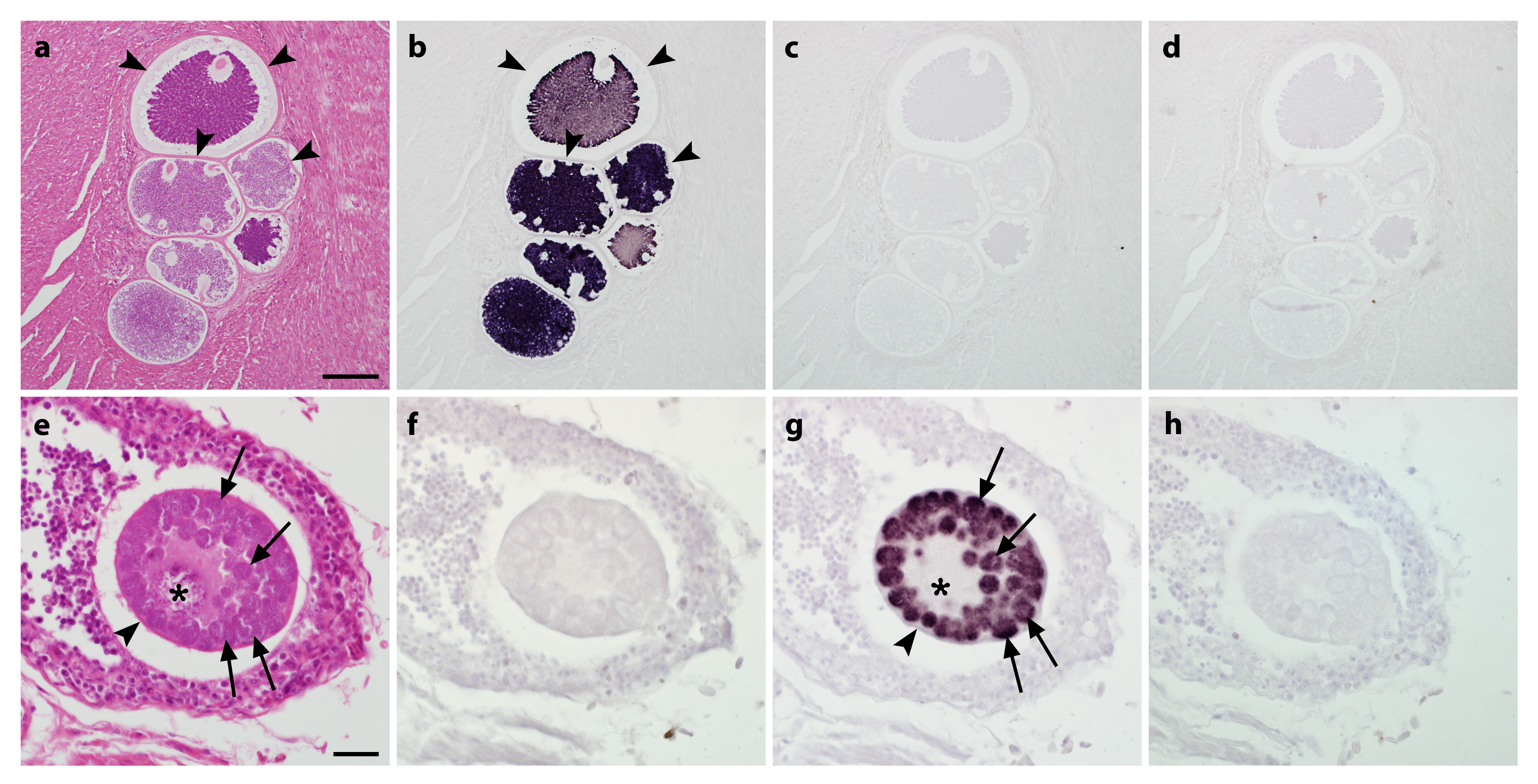

Supplement: Supplementary file 2 — Additional file 2: Figure S2. Megalomeronts of Haemoproteus minutus (a–d) and Leucocytocoon sp. (e–h) in histological sections from infected parakeets stained by haematoxylin-eosin (HE; a, e) and chromogenic in situ hybridization (CISH; b–d, f–h). Multiple megalomeronts of Haemoproteus minutus (TUPHI01), and Leucocytozoon cf. californicus (CIAE02), were observed in HE-stained sections of the cardiac muscle of Cyanoramphus novaezelandiae (a) and in the bursa of Fabricius of Brotogeris cyanoptera (c). CISH with probes for Haemoproteus (Parahaemoproteus) spp. (b, f), Leucocytozoon (Leucocytozoon) spp. (c, g) and Plasmodium spp. (d, h) indicated generic identity of parasites. Signals were confined to cytomeres (arrows) and merozoites of the parasites whereas host tissue structures like nucleus (asterisk) and capsule-like walls (arrowheads) around megalomeronts remained negative. Note numerous cytomeres (arrows) and a host cell nucleus present in the megalomeront of Leucocytozoon sp., but absent in megalomeronts of Haemoproteus parasites. Sections treated with probes not matching the parasites’ genera were negative (c, d, f, h). Scale-bars: a, 100 µm; e, 20 µm. [file 13071_2019_3536_MOESM2_ESM.tif]
